# Alteration of normal level of serum urate may contribute to decrease in estimated glomerular filtration rate decline in healthy Japanese men

**DOI:** 10.1080/0886022X.2021.1988969

**Published:** 2021-10-11

**Authors:** Akihiro Kuma, Kosuke Mafune, Bungo Uchino, Yoko Ochiai, Kazuhiko Enta, Akihiko Kato

**Affiliations:** a Kidney Center, Hospital of the University of Occupational and Environmental Health, Kitakyushu, Japan; b Blood Purification Unit, Hamamatsu University Hospital, Hamamatsu, Japan; c Department of Mental Health, Institute of Industrial Ecological Sciences, University of Occupational and Environmental Health, Kitakyushu, Japan; d Health Promotion Center, Yamaha Motor Co., Ltd, Iwata, Japan; e Health Care Center, Central Japan Railway Company, Nagoya, Japan

**Keywords:** Occupational health, chronic kidney disease, hyperuricemia, propensity score

## Abstract

**Introduction:**

Serum uric acid (SUA) levels have a linear relationship with the estimated glomerular filtration rate (eGFR). It is unclear whether further changes, subsequent to normal level of SUA can attenuate eGFR decline in a healthy population, so we aimed to determine the normal level of SUA that can contribute to preventing kidney dysfunction.

**Methods:**

In this retrospective cohort study from Japan, annual health checkup data from 2009 to 2014 was collected. After propensity score matching (1:1), data from 2,634 individuals with basal SUA ≤7.0 mg/dL (normal; mean age, 39 y; mean eGFR, 80.8 mL/min/1.73 m^2^) and 1,642 individuals with basal SUA >7.0 mg/dL (elevated; mean age, 42 y; mean eGFR, 75.0 mL/min/1.73 m^2^) were collected to determine the relationship between followed-up SUA level and the rate of change in eGFR.

**Results:**

In individuals with normal level SUA at baseline, the elevation of SUA (>7.0 mg/dL) accelerated eGFR decline compared to those with normal SUA levels at 5-year follow-up (−4.1 ± 9.6% vs −9.9 ± 9.0%, *p* < .0001). Digression of SUA level (≤7.0 mg/dL) reduced eGFR decline compared with persistent SUA level over 7.0 mg/dL (−1.5 ± 11.5% vs −7.0 ± 10.1, *p* < .0001). In multiple linear regression analysis, there was strong association between the rate of change in SUA and eGFR in individuals with basal SUA ≤7.0 and >7.0 mg/dL (standardized coefficient; −0.3348, *p* < .001 and −.2523, *p* < .001, respectively).

**Conclusion:**

Subsequent to normal level of SUA (under 7.0 mg/dL) may contribute to a decrease in eGFR decline in apparently healthy men.

## Introduction

Recent epidemiologic studies show a significant relationship between serum uric acid (SUA) levels and the development of kidney disease [1–3]. Hyperuricemia, defined as SUA >7.0 mg/dL in men, has a prevalence of 21.2% among men according to a U.S. study in 2008 [4]. According to a study of 21,475 healthy individuals, a slightly elevated SUA level (over 7.0 mg/dL) was associated with a two-fold increase in the risk for incident chronic kidney disease (CKD) [5].

Furthermore, in a study that included 13,338 participants with intact kidney function, the odds for incident CKD increased by 1.1 per 1.0 mg/dL increase in SUA after adjusting for age and several metabolic parameters [6].

In contrast, several studies have reported that decreased SUA levels were associated with a cessation in the progression of kidney dysfunction. In individuals whose SUA levels decreased to under 6.0 mg/dL from 9.75 mg/dL using allopurinol, a xanthine oxidase inhibitor, the serum creatinine levels increased more slowly compared with individuals from the placebo group during 12-months of observation [7]. In addition, a randomized controlled trial based in Japan found that the use of febuxostat, a non-purine selective inhibitor of xanthine oxidase, in individuals with CKD and hyperuricemia slightly changed serum creatinine levels [8]. Furthermore, in patients with CKD and hyperuricemia with an estimated glomerular filtration rate (eGFR) between 15–60 mL/min/1.73 m^2^, febuxostat slowed eGFR decline [9]. Finally, febuxostat has been shown to reduce SUA levels and have a favorable effect on eGFR, blood pressure, and albuminuria [10].

An SUA level under 6.0 mg/dL is the current target for CKD patients with an established diagnosis of gout, but this value is based on preventing gout, and appropriate SUA levels to reduce the progression of kidney disease have not been established in the literature [11]. In a Chinese study, individuals with CKD who took medication for hyperuricemia had mean SUA levels of 8.9–9.6 mg/dL [12]. The mean SUA was 7.8 mg/dL for a group treated with allopurinol during a randomized clinical trial in Spain, which indicated that the allopurinol treatment was a factor in reducing the progression of kidney disease [13]. In contrast, there is little evidence regarding the influence of hyperuricemia on kidney function in healthy populations. Therefore, this study aimed to describe the relationship between SUA and eGFR levels to recommend a normal level of SUA (≤7.0 mg/dL) for the early prevention of kidney disease in healthy young and middle-aged men.

## Materials and methods

### Study participants

**Figure 1.**
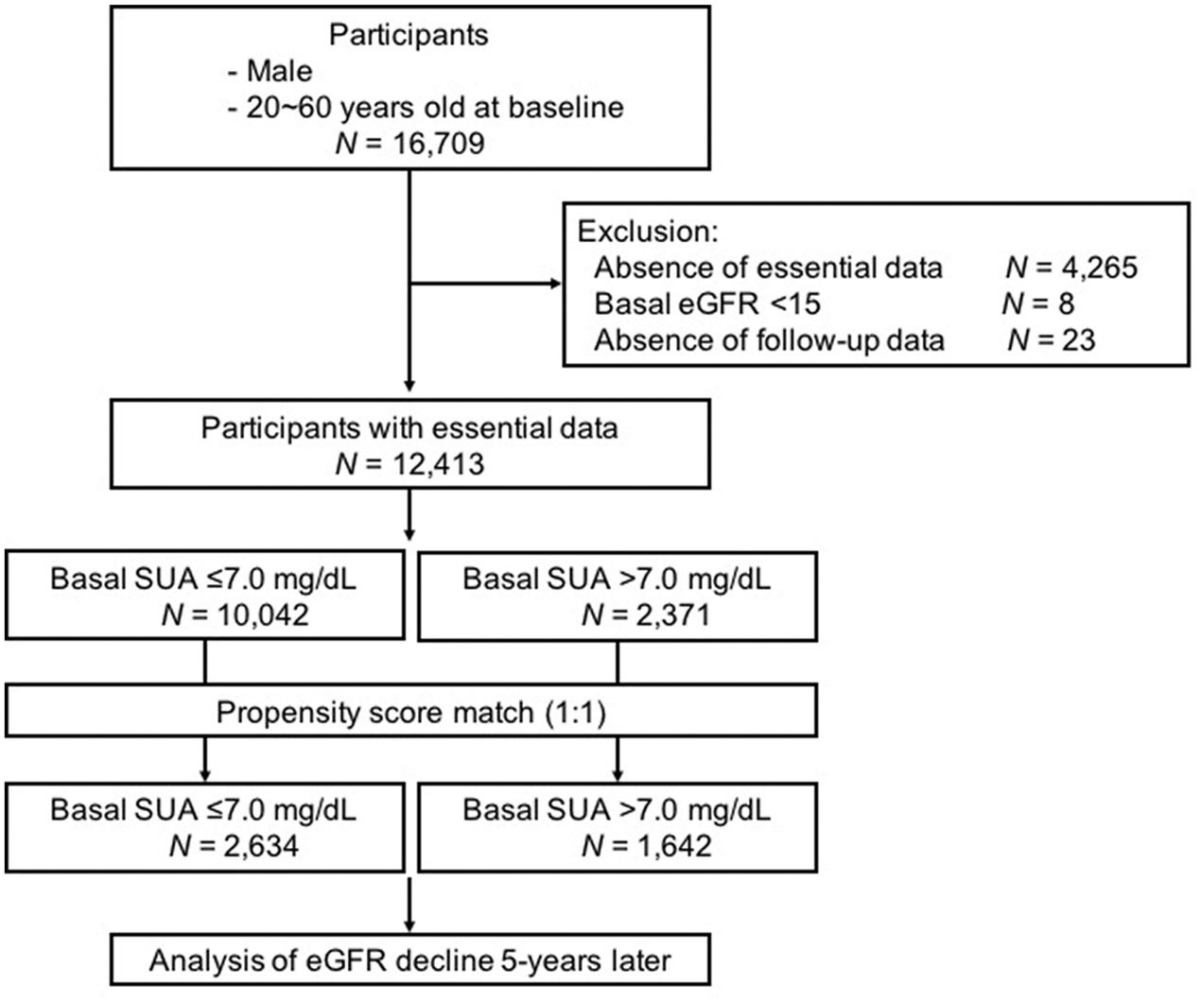
Flow chart of study participants. Essential data collected were estimated glomerular filtration rate (eGFR, ml/min/1.73 m^2^), body mass index, high-density lipoprotein cholesterol, triglycerides, serum uric acid (SUA), hemoglobin A1c, systolic/diastolic blood pressure, interview sheet, and follow-up data at 5 years from baseline.

This retrospective study was based on health checkup data and self-interview sheets of Japanese workers obtained from a couple of enterprises in Japan. We selected data of only male workers aged 20–60 years at baseline because female workers (*N* = 770) was considerably lesser than male workers (*N* = 16,709) in 2009. Annual health data from 2009 to 2014 checkups were collected and analyzed. Workers with missing essential data (*N* = 4,265), those with basal eGFR <15 mL/min/1.73 m^2^ (*N* = 8), and missing 5-year follow-up data (*N* = 23) were excluded (Figure. 1). For this retrospective cohort study, all participants were divided into two groups based on SUA level at baseline (normal, SUA level; ≤7.0 mg/dL; hyperuricemia, SUA >7.0 mg/dL) according to the Japanese guideline for the management of hyperuricemia and gout [14].

This study was approved by the ethical standards of the institutional committee (Hamamatsu University School of Medicine, N0. E15-289) and was performed in accordance with the 1964 Helsinki Declaration and its later amendments or comparable ethical standards. All individual participants were given the option to opt out of the study. Informed consent was waived by the ethics committee of Hamamatsu University School of Medicine due to the retrospective and anonymized study design. All methods were carried out in accordance with relevant guidelines and regulations.

### Health checkup data

At each checkup, blood pressure (BP), body height, and body weight were measured. Body mass index (BMI) was calculated as body weight (kg) divided by body height squared (m^2^). Blood and urine samples were tested by a private laboratory. eGFR was calculated using the following equation: eGFR (mL/min/1.73 m^2^) = 194 × serum creatinine^−1.094^ × age^−0.287^, according to previous studies [15]. The rate of change in eGFR and SUA levels were calculated using the following equation: rate of change in eGFR = (eGFR_2014_ – eGFR_2009_)/eGFR_2009_ and rate of change in SUA = (SUA_2014_ – SUA_2009_)/SUA_2009_. Information regarding smoking and alcohol consumption habits was acquired from a self-reported interview sheet. Smoking habit was defined by daily smoking. Alcohol consumption was defined as drinking alcohol 6 or 7 days a week.

### Statistical analyses

The rate of change in eGFR levels between the two groups after the 5-year follow-up period were compared. First, the Kolmogorov-Smirnov test was performed to assess the normal distribution of the parameters, and all clinical parameters showed normal distribution. The relationship between the rate of change in eGFR (dependent variance) and clinical parameters (independent variances) was analyzed by Pearson’s correlation coefficient (multiple linear regression). A student’s *t*-test or chi-square test was performed to compare the two groups. In this study, we accounted for the nonequivalence of this cohort *via* propensity score matching with logistic regression. A propensity score represents the predicted probability that a given participant will have elevated SUA (>7.0 mg/dL), and participants who had high-level SUA during a 5-year follow-up period were matched to control participants (SUA ≤7.0 mg/dL at 5-years follow-up period) with a greedy algorithm. The propensity score model included the interaction of age, basal eGFR, BMI, SUA, high-density lipoprotein cholesterol, triglycerides, hemoglobin A1c, systolic BP, habit of smoking, and habit of drinking alcohol.

Although this cohort was large (*N* = 16,708), we conducted 1:1 matching to avoid the possible bias of plural number-to-one matching. Propensity score matching was performed in two-classifications each: 1) SUA ≤7.0 mg/dL at baseline and 2) SUA >7.0 mg/dL at baseline. This process matched 1) 1,317 and 2) 821 individuals (99.8% and 53.4% of elevated SUA (>7.0 mg/dL) individuals, respectively) to the same number of control individuals (SUA ≤7.0 mg/dL) each (Tables 1 and 2). A covariate balance between matched elevated SUA individuals and control individuals was assessed with a two-tailed Student *t*-test and a chi-square test to confirm no statistical difference between the two groups.

**Table 1.** Baseline characteristics of participants with normal level of serum urate (≤7.0 mg/dL) at baseline.

	Unadjusted			Propensity score matched		
SUA 5-years later	≤7.0	>7.0	*P*-value	≤7.0	>7.0	*p* value
Participants, *N*	8,722	1,320		1,317	1,317	
Age, yr	38 (11)	39 (11)	.2917	38 (11)	38 (11)	.3907
eGFR, ml/min/1.73m^2^	82.7 (13.8)	80.9 (14.1)	<.0001	80.8 (12.9)	80.8 (14.1)	.9257
Body mass index, kg/m^2^	22.7 (3.1)	23.7 (3.3)	<.0001	23.5 (3.1)	23.7 (3.3)	.2231
SUA, mg/dL	5.5 (0.9)	6.4 (0.5)	<.0001	6.4 (0.5)	6.4 (0.5)	.9038
HDL-C, mg/dL	61 (14)	59 (15)	.0003	60 (14)	59 (15)	.3204
Triglycerides, mg/dL	110 (77)	128 (92)	<.0001	124 (102)	126 (79)	.6890
Hemoglobin A1c, %	5.3 (0.5)	5.3 (0.5)	.7784	5.3 (0.5)	5.3 (0.5)	.6589
Systolic BP, mmHg	122 (16)	125 (16)	<.0001	124 (16)	125 (16)	.3389
Smoking, *N* (%)	3,488 (40)	527 (40)	.9760	533 (40)	525 (40)	.7809
Alcohol, *N* (%)	2,078 (24)	422 (32)	<.0001	424 (32)	419 (32)	.8673

eGFR: estimated glomerular filtration rate; SUA: serum uric acid (mg/dl); HDL-C: high-density lipoprotein cholesterol; BP: blood pressure; Smoking: participants with daily habit of smoking; Alcohol: participants with habit of drinking alcohol six or seven days per week; Data: mean (standard deviation).

Propensity score matching (1:1) was performed by covariates of age, eGFR, HDL-C, triglycerides, hemoglobin A1c, systolic BP, smoking habitat, and alcohol consumption.

*p-*value was calculated by student *t-*test or chi square test.

**Table 2.** Baseline characteristics of participants with elevated serum urate (>7.0 mg/dL) at baseline.

	Unadjusted			Propensity score matched		
SUA 5-years later	≤7.0	>7.0	*P*-value	≤7.0	>7.0	*p* Value
Participants, *N*	834	1,537		821	821	
Age, yr	42 (10)	40 (10)	<.0001	42 (10)	43 (10)	.1268
eGFR, ml/min/1.73m^2^	75.1 (13.1)	76.8 (14.0)	.0036	75.3 (13.0)	74.7 (13.4)	.3905
Body mass index, kg/m^2^	24.5 (3.7)	24.9 (3.7)	.0144	24.6 (3.7)	24.5 (3.7)	.6387
SUA, mg/dL	7.7 (0.7)	7.9 (0.7)	<.0001	7.7 (0.7)	7.7 (0.5)	.2149
HDL-C, mg/dL	58 (15)	57 (14)	.0153	58 (15)	58 (15)	.6258
Triglycerides, mg/dL	152 (126)	160 (124)	.1235	153 (126)	153 (123)	.9540
Hemoglobin A1c, %	5.4 (0.5)	5.3 (0.4)	.0015	5.4 (0.5)	5.4 (0.5)	.9729
Systolic BP, mmHg	129 (17)	128 (16)	.1199	129 (17)	130 (17)	.6267
Smoking, *N* (%)	301 (36)	586 (38)	.3507	297 (36)	289 (35)	.7184
Alcohol, *N* (%)	302 (36)	505 (33)	.1024	294 (36)	307 (37)	.5387

eGFR: estimated glomerular filtration rate; SUA: serum uric acid (mg/dl); HDL-C: high-density lipoprotein cholesterol; BP: blood pressure; Smoking: participants with daily habit of smoking; Alcohol: participants with habit of drinking alcohol six or seven days per week; Data: mean (standard deviation).

Propensity score matching (1:1) was performed by covariates of age, eGFR, HDL-C, triglycerides, hemoglobin A1c, systolic BP, smoking habitat, and alcohol consumption.

*p-*value was calculated by student *t-*test or chi square test.

All analyses, except the propensity score matching, were performed using Stata 12SE software (Stata Co., College Station, TX, USA). SAS software, version 9.2 (SAS Institute Inc, NC, USA) was used for analysis of the propensity score matching. A *P*-value <0.05 was considered statistically significant.

## Results

### Participant characteristics at baseline

All participants were divided into two groups according to the basal SUA level of ≤7.0 (normal) or >7.0 mg/dL (elevated) first, then divided into two groups according to SUA level at 5-years later and basal characteristics were compared (Tables 1 and 2). In this cohort (*N* = 12,413), 21% of the participants were hyperuricemic at baseline. There were significant differences in the basal characteristics of eGFR, BMI, SUA, high-density lipoprotein cholesterol, triglycerides, habit of drinking alcohol, and systolic BP before propensity score matching in individuals with normal basal SUA. When the cohort was adjusted by propensity score matching, the comparison between the different basal characteristics was not significant (Table 1). In individuals with elevated SUA (>7.0 mg/dL), there were significant differences in the basal characteristics of age, eGFR, SUA, high-density lipoprotein cholesterol, and hemoglobin A1c before adjustment. After propensity score matching, these differences were not present (Table 2). Finally, 2,634 (basal SUA ≤7.0 mg/dL) and 1,642 (basal SUA >7.0 mg/dL) individuals were analyzed.

### Development of elevated SUA accelerated eGFR decline and improvement of elevated SUA slowed eGFR decline

**Figure 2.**
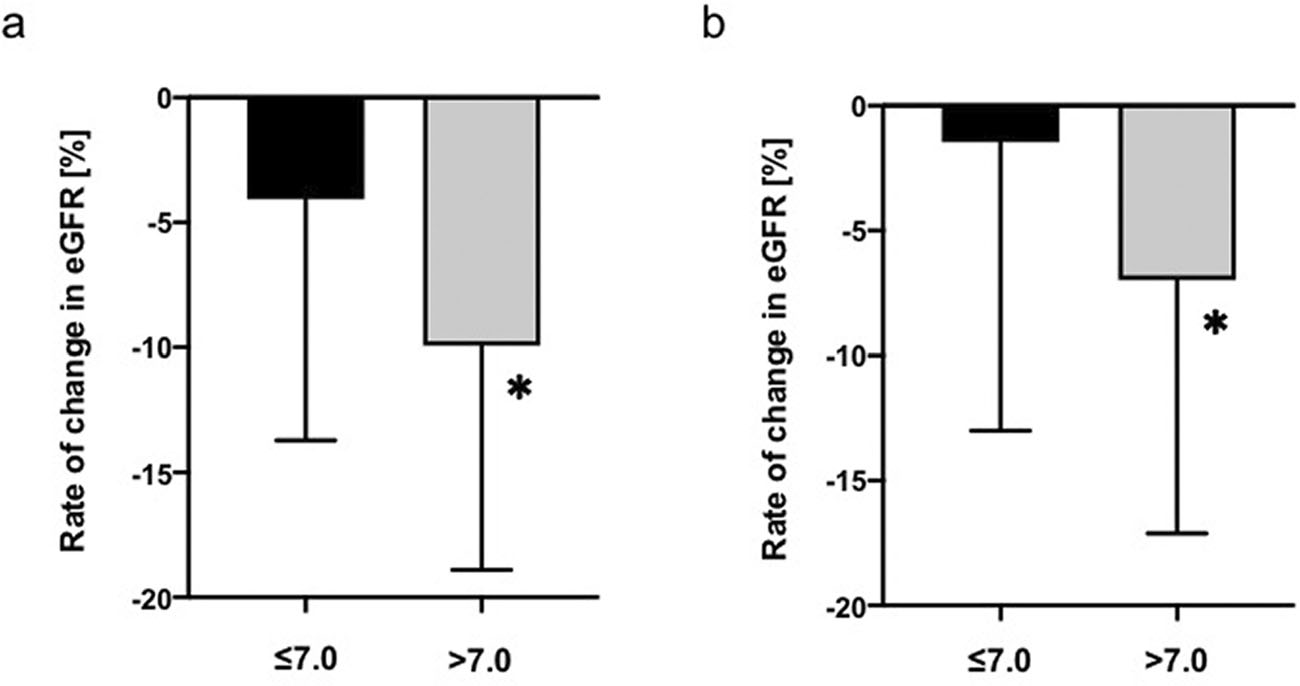
Comparison the rate of change in eGFR levels. (a) normal SUA (≤7.0 mg/dL) at baseline and (b) elevated SUA (>7.0 mg/dL) at baseline. Participants were divided to two groups categorized by SUA level at 5-year follow-up. Black bar: normal SUA ≤7.0 mg/dL and gray bar: elevated SUA >7.0 mg/dL in 2014. The rate of change in eGFR (%) = 100 × (eGFR_2014_ – eGFR_2009_)/eGFR_2009._ *p < 0.0001 vs normal SUA analyzed by unpaired *t* tests. eGFR, estimated glomerular filtration rate; SUA, serum uric acid.

After classification according to basal SUA, individuals were divided into two groups according to SUA levels at a 5-year follow-up. In individuals with normal basal SUA, the rate of change in eGFR for individuals with elevated SUA (>7.0 mg/dL) during the 5-year follow-up period was significantly lower than that for individuals with normal SUA (−4.1 ± 9.6% vs −9.9 ± 9.0%, *p* < 0.0001) (Figure. 2(a)). In contrast, in individuals with an improvement of SUA during the 5-year follow-up, the rate of change in eGFR decreased less than in individuals with elevated SUA at both points (−1.5 ± 11.5% vs −7.0 ± 10.1, *p* < 0.0001) (Figure. 2(b)).

### Change in SUA was strongly associated with a change in eGFR

We performed multiple linear regression analysis. In individuals with basal normal SUA, there was a significant negative relationship between the rate of change in SUA levels and eGFR (standardized coefficient = −0.3348, 95% confidence interval [CI]: −0.2242 to −0.1807) (Table 3). However, other characteristics were not significantly associated with a change in eGFR. In individuals with basal elevated SUA (>7.0 mg/dL), change in SUA and basal BMI and systolic BP were also significantly associated with a change in eGFR, but standardized coefficient of change in SUA was the largest (−0.2523, 95% CI: −0.2309 to −0.1585) (Table 4). Furthermore, there was not a large variance inflation factor in individuals with normal SUA and elevated SUA (Supplementary Table 1 and Supplementary Table 2).

**Table 3.** Multiple linear regression analysis with the rate of change in eGFR as dependent variable in basal normal SUA.

Variables	Standardized coefficient	Coefficient of regression	95% confidence interval (lower - upper)		*t* value	*p* Value
Age	0.0222	0.0002	−0.0002	0.0006	1.06	.291
Rate of change in SUA	−0.3348	−0.2025	−0.2242	−0.1807	−18.24	<.001
Body mass index	0.0049	0.0002	−0.0011	0.0014	0.23	.816
HDL-C	0.0396	0.0003	−0.0000	0.0006	1.86	.064
Triglycerides	−0.0112	−0.0000	−0.0001	0.0000	−0.55	.585
Hemoglobin A1c	0.0094	0.0018	−0.0060	0.0097	0.46	.646
Systolic blood pressure	−0.0095	−0.0001	−0.0003	0.0002	−0.45	.652
Smoking	−0.0096	−0.0019	−0.0093	0.0054	−0.51	.610
Alcohol	0.0390	0.0082	−0.0001	0.0164	1.94	.053

*N* = 2,634.

Model adjusted R^2^, 0.1143.

Model F, 38.74; *p* <.0001.

eGFR: estimated glomerular filtration rate; SUA: serum uric acid.

Rate of change in SUA = (SUA_2014_ – SUA_2009_) / SUA_2009_.

HDL-C: high-density lipoprotein cholesterol; Smoking: the daily habit of smoking; Alcohol: the habit of drinking alcohol six or seven days per week.

**Table 4.** Multiple linear regression analysis with the rate of change in eGFR as dependent variable in basal elevated SUA.

Variables	Standardized coefficient	Coefficient of regression	95% confidence interval (lower - upper)		*t* value	*p* Value
Age	0.0333	0.0004	−0.0002	0.0009	1.25	.231
Rate of change in SUA	−0.2523	−0.1947	−0.2309	−0.1585	−10.54	<.001
Body mass index	0.0953	0.0029	0.0012	0.0046	3.37	.001
HDL-C	0.0371	0.0003	−0.0001	0.0007	1.32	.187
Triglycerides	0.0224	0.0000	−0.0000	0.0001	0.85	.398
Hemoglobin A1c	−0.0549	−0.0121	−0.0237	−0.0005	−2.04	.041
Systolic blood pressure	−0.0687	−0.0005	−0.0008	−0.0001	−2.57	.010
Smoking	−0.0427	−0.0100	−0.0210	0.0011	−1.77	.076
Alcohol	0.0251	0.0058	−0.0060	0.0177	0.96	.335

*N* = 1,642.

Model adjusted R^2^, 0.0714.

Model F, 15.03; *p* <.0001.

eGFR: estimated glomerular filtration rate; SUA: serum uric acid.

Rate of change in SUA = (SUA_2014_ – SUA_2009_) / SUA_2009_.

HDL-C: high-density lipoprotein cholesterol; Smoking: the daily habit of smoking; Alcohol: the habit of drinking alcohol six or seven days per week.

## Discussion

This retrospective study analyzed health data based on over 12,000 working men in Japan, and over 4,000 individuals who were selected by propensity score matching were analyzed. Our results indicate that elevation of SUA accelerated eGFR decline and decrease of SUA below 7.0 mg/dL slowed eGFR decline. Furthermore, the rate of eGFR decline was significantly associated with a change in SUA regardless of basal SUA level. These findings may contribute to kidney dysfunction prevention in apparently healthy men.

A high level of SUA is associated with endothelial dysfunction induced by reactive oxygen species (ROS) [16,17]. Xanthine oxidase, a member of the ROS family, reacts with xanthine and produces uric acid and superoxide anions. Oxidative stress, endothelial dysfunction, and activation of renin-angiotensin system lead to kidney dysfunction. In addition, UA crystals induce interstitial inflammation in the kidney tubules [18,19].

We used 7.0 mg/dL of SUA as the cutoff point, because hyperuricemia is defined as >7.0 mg/dl of SUA in most countries, including Japan [4,14]. Furthermore, our previous investigation showed that 6.8 mg/dl of SUA (sensitivity 0.4368, specificity 0.6898, Youden’s index 0.1266) was an appropriate cutoff value for incident CKD in our cohort by receiver operating characteristic curve analysis (Supplementary Figure 1). Interestingly, urate solubility point is 6.8 mg/dL [20]. Hence, we believe SUA level of 7.0 mg/dL is an appropriate point to avoid proceeding of kidney dysfunction in apparently healthy population, similar to our cohort.

Several epidemiological studies have shown a relationship between hyperuricemia and progression of CKD [21–23], but the study populations included individuals with CKD stage 3 or 4. One analysis of 14,399 Chinese individuals with normal eGFR (about 100 mL/min/1.73 m^2^) also showed the relationship between hyperuricemia, albuminuria, reduced eGFR, and CKD [24], but it was unable to investigate cause and effect, because it was a cross-sectional study. In our cohort, normal level of SUA at a 5-year follow up may contribute to the reduction of eGFR decline in individuals with over 60 mL/min/1.73 m^2^ of eGFR at baseline (Supplementary Figure 2).

The present study confirmed the beneficial effect of lowering SUA levels in individuals with CKD (Supplementary Figure 3). In a randomized clinical trial, 2-year treatment with allopurinol slowed the rate of eGFR decline by 50% and reduced cardiovascular disease even in patients with CKD stage 3 or 4 at baseline [13]. In addition to allopurinol, a 6-month treatment with febuxostat was shown to reduce SUA from 9.0 mg/dL to 5.2 mg/dL and there were fewer participants with ≥10% decrease in eGFR (basal eGFR; 31.5 mL/min/1.73 m^2^) in the treatment group than in the placebo group [9]. Evidence from interventional clinical studies regarding the treatment of hyperuricemia with urate-lowering medication in participants without CKD is lacking. However, the present study showed that downregulation of elevated SUA (>7.0 mg/dL) in individuals without CKD was significantly associated with a reduction in eGFR decline due to the longitudinal approach to individual-level data collection (Supplementary Figure 2(b)).

In contrast, in the Controlled Trial of Slowing of Kidney Disease Progression from the Inhibition of Xanthine Oxidase, which was a randomized-controlled trial with 363 CKD patients in Australia and New Zealand, allopurinol did not slow the decrease in eGFR in patients with CKD stage 3 or 4 at the 104-week follow-up, although the average SUA level decreased to under 6.0 mg/dL in the allopurinol group [25]. However, in that study, the allopurinol intervention trial had mean SUA level, 8.2 mg/dL, which was rather different than commonly used intervention threshold (≥ 10 mg/dL) in most interventional study. Therefore, the effect of allopurinol might be weaken in that study. Even so, we think the management of SUA is important before the development of incident CKD. Although SUA level under 7.0 mg/dL indicates no hyperuricemia, SUA less than <5.0 mg/dL maybe better than high-normal SUA (5.0 to ≤7.0 mg/dL) for kidney function (Supplementary Figure 4). We need further investigations to determine a better cutoff point to avoid CKD development in the future.

Previous several meta-analyses showed allopurinol or febuxostat were associated with the lower risk of eGFR decline [26,27]. On the other hand, some studies reported no effect of febuxostat on eGFR decline with asymptomatic hyperuricemia in CKD stage 3 patients [28,29]. In addition, recent systematic review has showed evidence that asymptomatic hyperuricemia should be treated under limited conditions: persistent SUA level higher than 13 mg/dL (men) or 10 mg/dL (women) and urinary excretion of uric acid exceeding 1,100 mg daily [30,31]. For individuals of asymptomatic hyperuricemia like our cohort, there have been no sufficient evidence to treat hyperuricemia. Therefore, we think further investigation about treatment for asymptomatic hyperuricemia will be necessary in the future.

This study had some major limitations to consider. First, women were excluded due to the lack of robust data since there were fewer female employees (*N* = 770). Second, this study used health checkup data and self-interview sheets, which were filled up before collecting them. Thus, we could not get information regarding medications (anti-hyperuricemic drugs, losartan, or diuretics) and could not investigate the influence of these medicines on eGFR decline. Third, we selected participants from our cohort using the propensity score matching (1:1) to avoid the possible bias of participants’ background. However, influence of kidney condition and non-homogeneity could not be completely eliminated from the statistical analyses.

In the present study, we found that downregulation of elevated SUA reduced eGFR decline during a 5-year follow-up period in apparently healthy individuals regardless of CKD at baseline. Our findings may contribute to a new standard of SUA management to reduce the progression of kidney dysfunction in apparently healthy populations.

## Supplementary Material

Supplemental Material

## Data Availability

The datasets generated during and/or analyzed during the current study are not publicly available due to the rule of third parties which provided data to our facility but are available from corresponding author on reasonable request.
