# Supplementary material for: Alteration of normal level of serum urate may contribute to decrease in estimated glomerular filtration rate decline in healthy Japanese men
Source: Ren Fail. 2021 Oct 11;43(1):1408–15. doi: 10.1080/0886022X.2021.1988969 (PMC8510623; doi:10.1080/0886022X.2021.1988969)
Supplement: Supplemental Material [file IRNF_A_1988969_SM3423.pdf]

Supplementary Table 1.
Variance inflation factor in multiple linear regression analysis in basal normal SUA

Variables	Variance inflation factor
Age	1.32
Rate of change in SUA	1.00
Body mass index	1.33
HDL-C	1.35
Triglycerides	1.24
Hemoglobin A1c	1.24
Systolic blood pressure	1.32
Smoking	1.05
Alcohol	1.20

SUA; serum uric acid
HDL-C; high-density lipoprotein cholesterol
Smoking; the daily habit of smoking
Alcohol; the habit of drinking alcohol six or seven days per week

Supplementary Table 2.
Variance inflation factor in multiple linear regression analysis in basal elevated SUA

	Variance inflation factor
Age	1.26
Rate of change in SUA	1.01
Body mass index	1.41
HDL-C	1.40
Triglycerides	1.24
Hemoglobin A1c	1.28
Systolic blood pressure	1.27
Smoking	1.02
Alcohol	1.20

SUA; serum uric acid
HDL-C; high-density lipoprotein cholesterol
Smoking; the daily habit of smoking
Alcohol; the habit of drinking alcohol six or seven days per week

Supplementary Figure 1.

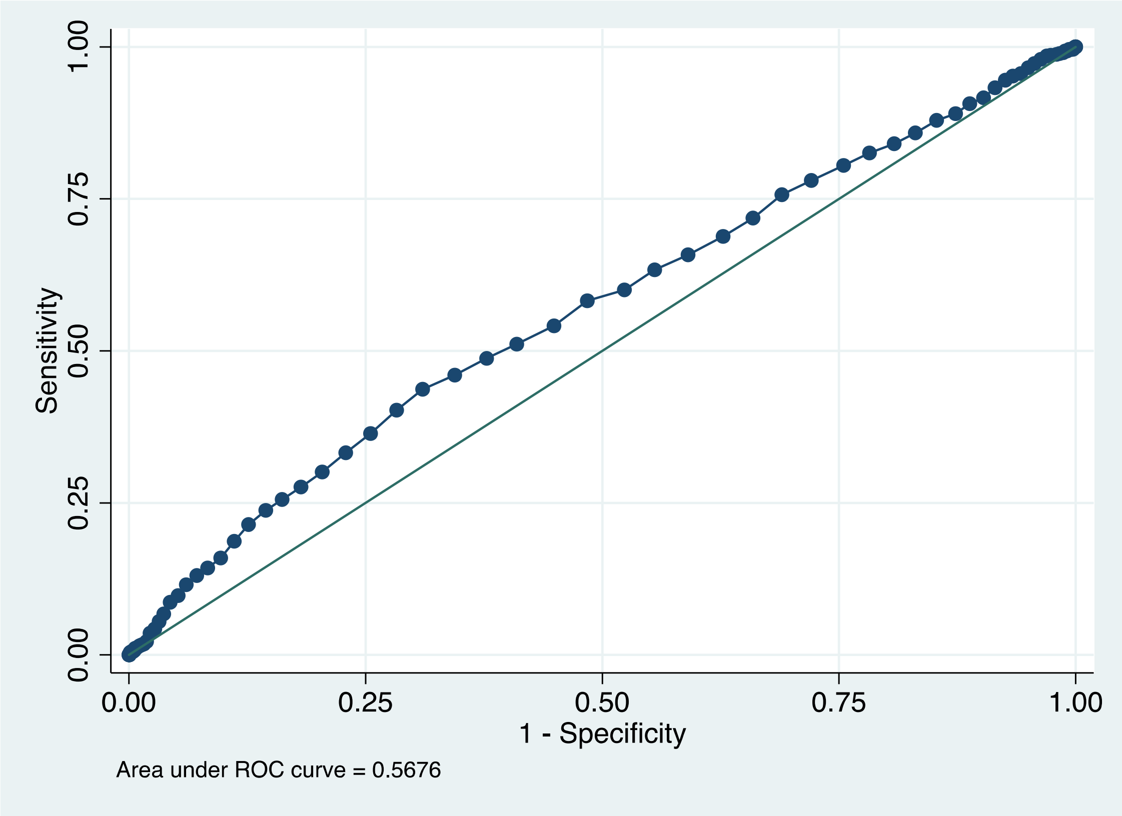

Receiver operating characteristic (ROC) curve analysis for CKD incident according to serum level of uric acid

ROC curves were calculated using the rate of incident CKD (eGFR <60 ml/min/1.73m^2) at 5-year follow-up and serum level of uric acid at baseline. *N* = 8,207. ROC area: 0.5676 (95% confidence interval: 0.5449 to 0.5902). CKD; chronic kidney disease, eGFR; estimated glomerular filtration rate.

Supplementary Figure 2.

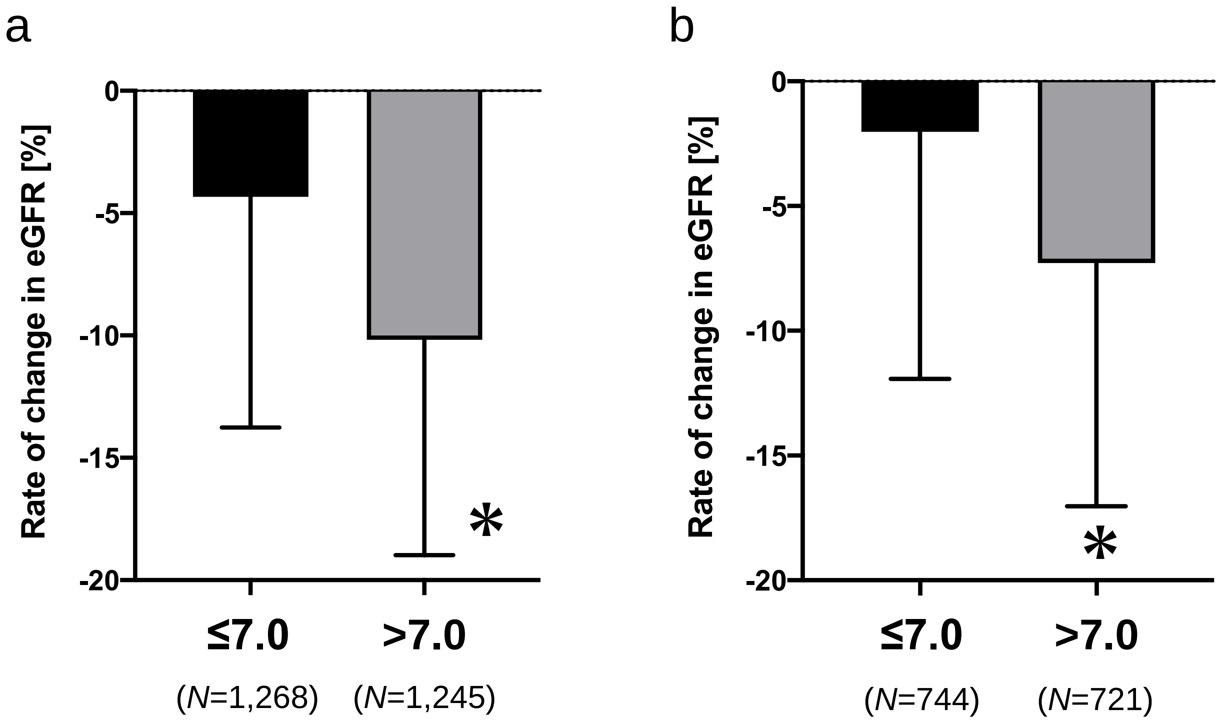

Comparison the rate of change in eGFR levels in individuals with eGFR ≥60 ml/min/1.73m^2 at baseline
(a) normal SUA (≤7.0 mg/dL) at baseline and (b) elevated SUA (>7.0 mg/dL) at baseline. Participants were divided to two groups categorized by serum uric acid (SUA) level at 5-year follow-up. Black bar: normal SUA ≤7.0 mg/dL and gray bar: elevated SUA >7.0 mg/dL in 2014. The rate of change in eGFR (%) = 100 x ($eGFR_{2014}$ – $eGFR_{2009}$) / $eGFR_{2009}$. Rate of change in eGFR were (a) -4.3 ± 9.4% (black bar) and -10.2 ± 8.8% (gray bar) and (b) -2.0 ± 9.9% (black bar) and -7.3 ± 9.8% (gray bar).
**P* <0.0001 vs normal SUA analyzed by unpaired *t* tests. eGFR; estimated glomerular filtration rate, SUA; serum uric acid.

Supplementary Figure 3.

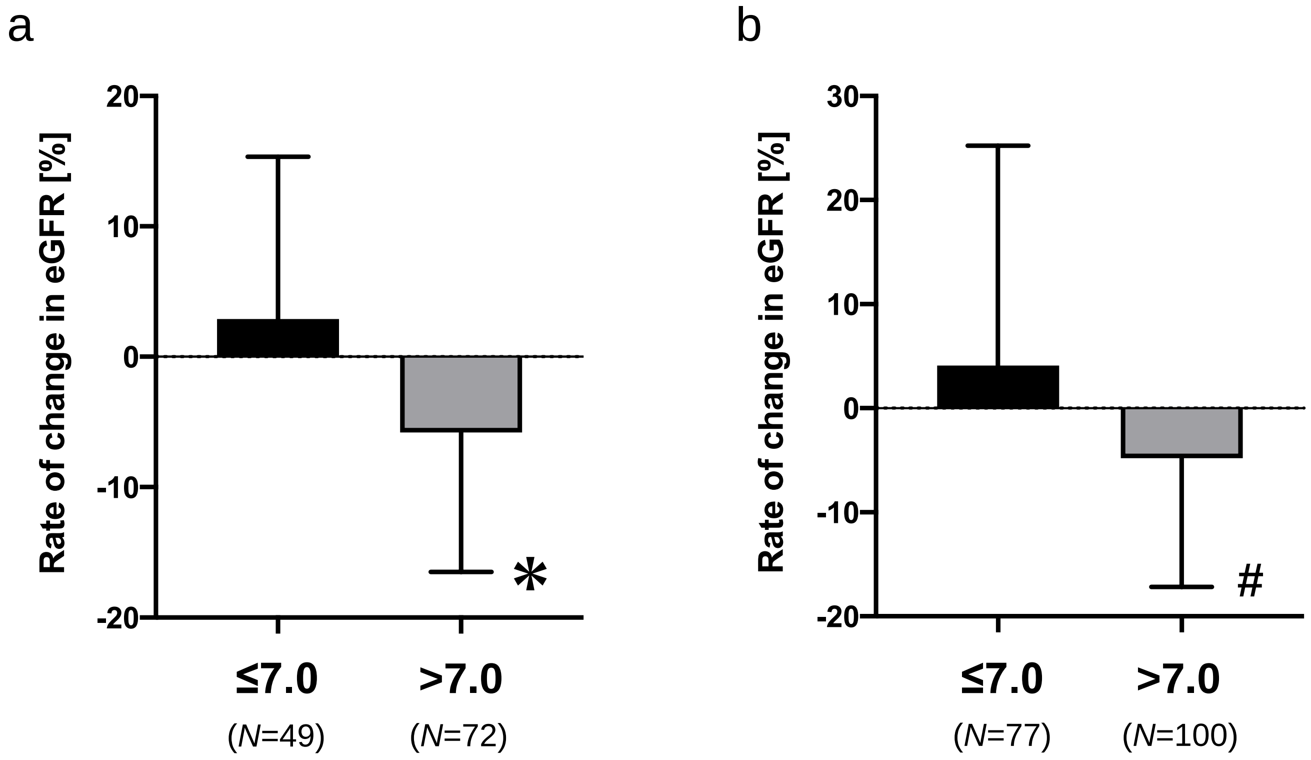

Comparison the rate of change in eGFR levels in individuals with eGFR <60 ml/min/1.73m^2 at baseline
(a) normal SUA (≤7.0 mg/dL) at baseline and (b) elevated SUA (>7.0 mg/dL) at baseline. Participants were divided to two groups categorized by SUA level at 5-year follow-up. Black bar: normal SUA ≤7.0 mg/dL and gray bar: elevated SUA >7.0 mg/dL in 2014. The rate of change in eGFR (%) = 100 x ($eGFR_{2014}$ – $eGFR_{2009}$) / $eGFR_{2009}$. Rate of change in eGFR were (a) 2.9 ± 12.5% (black bar) and -5.9 ± 10.7% (gray bar) and (b) 4.1 ± 21.1% (black bar) and -4.8 ± 12.4% (gray bar).
$^{*}P$ =0.0001 and $^{\#}P$ =0.0006 vs normal SUA analyzed by unpaired *t* tests. eGFR; estimated glomerular filtration rate, SUA; serum uric acid.

Supplementary Figure 4.

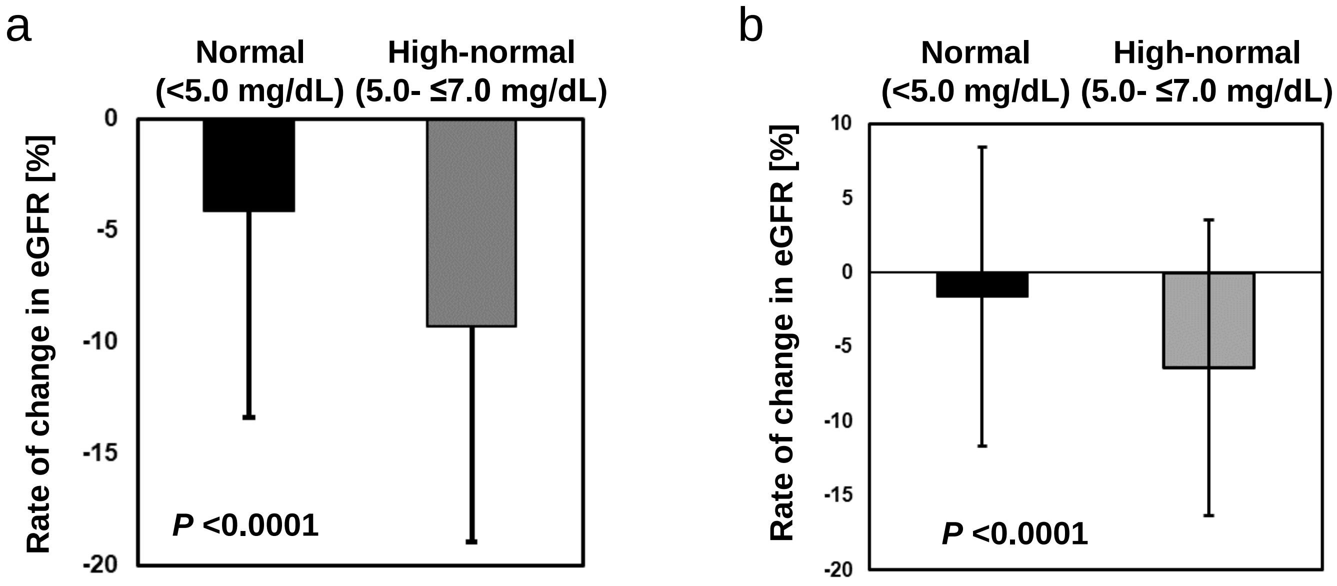

Comparison the rate of change in eGFR levels in individuals categorized by SUA less than <5.0 mg/dl or 5.0 - ≤7.0 mg/dL at baseline

After propensity score matching, rate of change in eGFR was compared between normal SUA and high-normal SUA level 5-year follow-up. (a) normal SUA (less than <5.0 mg/dL) at baseline (*N* = 560 each group) and (b) high-normal SUA (5.0 - ≤7.0 mg/dL) at baseline (*N* = 523 each group). Participants were divided to the two groups categorized by SUA level at 5-year follow-up. Black bar: normal SUA <5.0 mg/dL and gray bar: high-normal SUA 5.0 - ≤7.0 mg/dL in 2014. The rate of change in eGFR (%) = 100 x ($eGFR_{2014}$ – $eGFR_{2009}$) / $eGFR_{2009}$. *P* <0.0001: normal SUA vs high-normal SUA analyzed by unpaired *t* tests. eGFR; estimated glomerular filtration rate, SUA; serum uric acid.
